# A Long Way Is Ahead of Prediction of Menopause!

**DOI:** 10.5812/ijem.6836

**Published:** 2012-06-30

**Authors:** Fahimeh Ramezani Tehrani

**Affiliations:** 1 Reproductive Endocrinology Research Center, Research Institute for Endocrine Sciences, Shahid Beheshti University of Medical Sciences, Tehran, IR Iran

**Keywords:** Menopause, Fertility

Menopause, an important reproductive milestone, is associated with complete cessation of fertility. There is considerable individual variation in the age of onset of menopause; its average is 51 years but varies from 40 to 60 years and about 10% of women reach menopause by the age of 45 years (1).

Over the past two decades, precise prediction of age at menopause for individual women has attracted a lot of interest. Nowadays many women postpone their childbearing but with a lot of anxiety about the amount of time remains before reaching menopause. The prediction of age at menopause would possibly help these women to make a reasonable decision regarding their family planning.

Various endocrinological and sonographic markers have been used to predict age at menopause and to assess ovarian reserve status (2, 3). In spite of subtle changes in endocrine regulation of ovarian function with advancing age the majority of these markers are not clinically useful for prediction of menopause. The majority of changes on these factors is happening during the last years of reproduction and is not appropriate for an early prediction; furthermore they have significant variation from one menstrual cycle to another as a result a single measurement does not provide a reliable and valid assessment of reproductive status (4).

There are recent clinical insights into Anti Mullerian Hormone (AMH) as a promising biomarker for assessment of the ovarian follicular status (5). Anti-Mullerian hormone (AMH) is a glycoprotein that belongs to the transforming growth factor β superfamily. It is mainly expressed by the Sertoli cells in the fetal testis and by the Granolosa cells of ovarian follicles (6). The physiological role of AMH in female reproduction is not fully described however animal studies demonstrated its role in both the inhibition of primordial to primary follicle growth and also in the follicular responsiveness to follicular stimulating hormone (FSH) (7, 8).

AMH has several characteristics that make it theoretically as a suitable biologic marker for ovarian aging. AMH is secreted exclusively in ovarian follicles and gradually decreases with increasing age (4, 9). It is independent of the menstrual cycle and only minor fluctuations in serum concentrations have been observed during the normal menstrual cycle that is consistent with the continuous non cyclic growth of small follicles (9). Its level remains almost constant from one cycle to another and has a high intra class correlation coefficient as a result only one measurement will provide a reliable estimate of its mean in each individual woman (10, 11).

Over the past decade much effort has been put into a precise prediction of individual age at menopause based on the serum concentration of AMH (12-14). There are a limited number of cohort studies that used various statistical modeling to provide a tool for clinicians for menopausal age’s prediction (14, 15). However the existing cohorts have the limitation of not including women from early reproductive age and not long-enough duration of follow ups.

It seems that AMH has the potential capacity for assessment of ovarian ageing but we have a long way ahead to use it as a clinical tool for simple and reliable prediction of age at menopause. Several large longitudinal study representative of the general population of various ethnicities with a long-enough duration, starting with women in their early reproductive period with a comprehensive assessment of their clinical, biochemical and genetic profile are urgently needed.
